# A non-exhaustive survey revealed possible genetic similarity in mitochondrial adaptive evolution of marine fish species in the northwestern Pacific

**DOI:** 10.3897/zookeys.974.55934

**Published:** 2020-10-07

**Authors:** Linlin Zhao, Tianzi Wang, Fangyuan Qu, Zhiqiang Han

**Affiliations:** 1 The First Institute of Oceanography, Ministry of Natural Resources, Qingdao, China The First Institute of Oceanography, Ministry of Natural Resources Qingdao China; 2 Fishery College, Zhejiang Ocean University, Zhoushan, China Zhejiang Ocean University Zhoushan China

**Keywords:** Cytochrome *b* gene, genetic similarity, marine fish, Northwestern Pacific, population genetics

## Abstract

Mitochondrial coding genes involved in the oxidative phosphorylation pathway play vitally important roles in energy production and thermal adaptation. Investigating the underlying molecular mechanism of mitochondrial adaptive evolution is crucial for understanding biodiversity and ecological radiation. In this study, we collated population genetic studies of marine fish species in the northwestern Pacific based on mitochondrial cytochrome *b* gene sequences, to investigate whether similar patterns could be detected in mitochondrial adaptive evolution. After filtering, nine studies containing eight marine fish species (*Ammodytes personatus*, *Boleophthalmus pectinirostris*, *Larimichthys polyactis*, *Mugil cephalus*, *Pampus argenteus*, *Platycephalus* sp.1, *Sebastiscus marmoratus*, and *Trachidermus fasciatus*) belonging to eight different families were retained. Multiple codon-based approaches were used to identify potential sites under selection in each species. By comparison, our results showed that the posterior part of the mitochondrial cytochrome *b* gene (particularly codon 372 and its neighboring sites) seemed to be involved in the adaptive evolution process, suggesting potential genetic similarity among distantly related species. We also summarized four types of adaptive patterns in the reviewed species, and suggest that the level of genetic differentiation and mitochondrial adaptive evolution might be correlated. Further studies are needed to confirm such relationship by detecting RNA-level evidence and investigating more species and samples.

## Introduction

Understanding adaptive evolution of marine organisms is a focus topic in evolutionary biology, and can also provide essential information for fishery management and conservation (Postma and van Noordwijk 2005; Palsbøll et al. 2007). In marine realm, due to a lack of physical barriers, most environmental factors are relatively homogeneous compared to the terrestrial environment (Gleason and Burton 2016), and temperature is thought to be the most significant variable among different latitudinal gradients (Silva et al. 2014; Xu et al. 2017). Marine species with a wide latitudinal distribution normally experience various degrees of thermal tolerance across the distribution ranges, which could result in thermally adaptive evolution. In turn, genetic variations resulting from thermal adaptation could help organisms adapt to a local environment, and simultaneously contribute to genetic differentiation among geographically distant populations (Silva et al. 2014). Divergent local adaptation may generate barriers to population connectivity and ultimately lead to ecological speciation (Schluter and Rambaut 1996; Momigliano et al. 2017). It can be assumed that adaptive sites would be fixed among populations during the adaptation process, resulting in and maintaining genetic differentiation. Consequently, intraspecific adaptive evolution could be relatively easy to detect when population differentiation is weak (Lamichhaney et al. 2017). When strong genetic differentiation was maintained, strict purifying selection and relaxed positive selection might buffer the adaptation differentiation scenario (Nosil et al. 2009; Forester et al. 2016).

Given the relatively fast mutation rate, mitochondrial cytochrome *b* (cytb) and the non-coding control region are the most two mitochondrial genes investigated in population genetics of marine fish species (Guo et al. 2004). In the mitochondrial genome, protein coding genes involved in the oxidative phosphorylation (OXPHOS) pathway play vital important roles in energy production and respiration (Ruiz-Pesini et al. 2004; Sun et al. 2011). However, despite the important function of energy production and thermoregulation, mitochondrial DNA has been considered as an evolutionary bystander in population genetics and phylogenetics (Ballard and Pichaud 2014). Until now, limited numbers of studies have shown evidence for mitochondrial adaptive evolution in marine organisms (but see Sun et al. 2011; Caballero et al. 2015; Jacobsen et al. 2016). Among them, species-specific patterns were detected in mitochondrial adaptive evolution, suggesting varied genetic mechanisms. In contrary, our previous studies consistently revealed that adaptive sites were detected in the posterior part of mitochondrial cytb gene (Xu et al. 2017, 2018), to some extent suggesting potential genetic similarity in adaptive evolution of cytb gene. To confirm such potential similarity, here we collated population genetic studies of marine fish species in the northwestern Pacific based on mitochondrial cytb gene sequences and detect genetic adaptive sites by using population-based approaches. Our findings provide supplementary information for the evolutionary biology of marine organisms.

## Methods

### Literature collation

Considering that our major research field is population genetics of marine fish in the northwestern Pacific, we searched population genetics papers based on mitochondrial cytb gene sequences by using the search terms “population genetics”, “cytochrome *b* or cytb”, “marine fish species” and “northwestern Pacific” in Google Scholar and China National Knowledge Infrastructure (CNKI) literature databases. Our previous studies revealed few genetic variations in the front half of the cytb gene (Xu et al. 2017, 2018), suggesting the front part of the cytb gene is relatively conserved and unsuitable for population genetic analyses. Given that, papers only analyzing the front part of cytb gene sequences were excluded. In addition, papers with unreleased sequence data were also excluded. After filtering, nine papers containing eight marine fish species (*Ammodytes personatus* Girard, 1856; *Boleophthalmus pectinirostris* (Linnaeus, 1758); *Larimichthys polyactis* (Bleeker, 1877); *Mugil cephalus* Linnaeus, 1758; *Pampus argenteus* (Euphrasen, 1788); *Platycephalus* sp. 1 (sensu Nakabo, 2002); *Sebastiscus marmoratus* (Cuvier, 1829); and *Trachidermus fasciatus* Heckel, 1837) were retained (Table 1). The cytb gene sequences were downloaded from GenBank. We also downloaded cytb gene sequences of *Larimichthys crocea* (Richardson, 1846) (GenBank accessions: EU346914–346932), the sister species of *L. polyactis*, to investigate interspecific mitochondrial adaptive evolution.

### Analyses of adaptive evolution

For each of the nine species, sequences were aligned using MAFFT method in Unipro UGENE v1.12.0 software (Okonechnikov et al. 2012). MEGA 6.0 software (Tamura et al. 2013) was applied to detect fixed adaptive sites based on branches of neighbor-joining topologies. For natural selection tests, the best fitting substitution model was firstly tested using the Model Selection algorithm implemented in HYPHY package on the DataMonkey server (Kosakovsky Pond et al. 2005; Delport et al. 2010) and used in the following selection tests on the DataMonkey server. To detect putatively adaptive sites, six codon-based selection tests were applied for selection inference to the data: CODEML (Yang 2007), SLAC (single likelihood ancestor counting, Kosakovsky Pond and Frost 2005), FEL (fixed effects likelihood, Kosakovsky Pond and Frost 2005), IFEL (internal fixed effects likelihood, Kosakovsky Pond et al. 2006), FUBAR (fast unconstrained Bayesian approximation, Murrell et al. 2013) and MEME (mixed effects model of evolution, Murrell et al. 2012) algorithms. Among them, CODEML algorithm was analyzed in PAML package (Yang 2007) and SLAC, FEL, IFEL, FUBAR and MEME algorithms were implemented in HYPHY package on the DataMonkey server. All these methods were applied to prevent against our results being an artifact of a particular methodology or a set of assumptions. Positively selected sites detected in at least two tests were considered to be positively adaptive sites. Significance was assessed by posterior probability (pp) >0.9 (FUBAR) and P-value <0.05 (SLAC, FEL, IFEL and MEME).

**Table 1.** General information of reviewed marine species in this study.

Order	Family	Species	Latitudinal range	GenBank accession	NH^2^	Reference
Perciformes	Sciaenidae	*Larimichthys polyactis*	39.8–27.2N	FJ609001–609137; JN601196–601289	231	Huang 2011; Wu et al. 2009
	Gobiidae	*Boleophthalmus pectinirostris*	34.8–20.8N	KF384522–384638, KF415515	118	Chen et al. 2015
	Ammodytidae	*Ammodytes personatus*	45.5–35.9N	MK112908–113077	170	Deng et al. 2019
	Stromateidae	*Pampus argenteus*	19.2–11.6N^1^	JF790202–790259, KJ630414–630460	105	Sun et al. 2012
Scorpaeniformes	Sebastidae	*Sebastiscus marmoratus*	37.9–21.5N	KX374371–374400, KX722503–722509	30	Xu et al. 2017
	Platycephalidae	*Platycephalus* sp.1	35.5–21.4N	MG913953–913986	34	Xu et al. 2018
	Cottidae	*Trachidermus fasciatus*	39.8–30.4N	JX079997–080027, KC701150–701194	76	Gao et al. 2013
Mugiliformes	Mugilidae	*Mugil cephalus*	32.7–22.0N	EU083809–083903	95	Ke et al. 2009

Note: ^1^ individuals under accessions KJ630414–630460 were not included in this range. ^2^ NH: number of haplotypes.

## Results and discussion

A total of 987 mitochondrial cytb complete gene sequences were downloaded from the GenBank database for intraspecific analyses (Table 1). We also downloaded 19 *Larimichthys crocea* sequences for interspecific comparison with *L. polyactis*. No positively and fixed adaptive sites were detected in *L. polyactis* and *B. pectinirostris* based on the selection tests. Conversely, positively and/or fixed adaptive sites were detected in the remaining six species, including both positively and fixed adaptive sites in *P. argenteus* (Table 1). Previous studies revealed that the population differentiation was weak in *L. polyactis*, due to a high migration ability (Xiao et al. 2009). High migration ability could facilitate population connectivity and lead to population panmixia, especially in marine realms (Moody et al. 2015). Adaptive evolution of *L. polyactis* might be impeded by the high gene flow among populations. For *B. pectinirostris*, population differentiation mainly resulted from 13 non-synonymous substitutions (sites 102, 168, 219, 249, 330, 429, 468, 489, 531, 592, 735, 828, 846). As a result, no positively or fixed adaptive sites were detected in *B. pectinirostris*. *Pampus argenteus* was the only species with both positively and fixed adaptive sites. Considering the relatively wide sampling range (samples from South China Sea, the Bay of Bengal, and the Arabian Sea), physical barriers such as the Malaya and India peninsulas might force *P. argenteus* to split into different ecotypes (Sun et al. 2012). The same results were also detected in *Engraulis encrasicolus* (Silva et al. 2014). Based on the cytb haplotype sequences in Silva et al. (2014), we identified codon 368 as the positive fixed adaptive site among *E. encrasicolus* populations. Considering the trans-equatorial sampling range, *E. encrasicolus* might also split into different ecotypes (Le Moan et al. 2016). Positive and fixed adaptive sites contribute to and largely maintain intraspecific differentiation (Pearse et al. 2014; MacPherson and Nuismer 2017; Lai et al. 2019). Our results also revealed that positively adaptive sites could be detected when genetic differentiation was weak, and that fixed adaptive sites can be detected when strong genetic differentiation occurred (Table 2). Such a conclusion is consistent with different stages of ecological speciation (Rundle and Nosil 2005; Schluter and Conte 2009; Schluter 2009). Under this process, adaptive evolution caused by natural selection acts in contrasting directions between distinct environments, which drives the fixation of different alleles each advantageous in one environment but not in the other. Population differentiation would strengthen as they accumulate a different series of mutations (Schluter and Conte 2009).

**Table 2.** Results of adaptive evolution analyses of reviewed marine species in this study.

Species	Model	Genetic background	Purifying site^1^	Positively adaptive site^2^	Fixed adaptive site^3^
*Larimichthys polyactis*	012032	Weak genetic differentiation	121 codons	Undetected	Undetected
*Sebastiscus marmoratus*	010010	Weak genetic differentiation	Codon 287	Codon 372	Undetected
*Platycephalus* sp.1	010000	Weak genetic differentiation	12 codons	Codon 314	Undetected
*Trachidermus fasciatus*	010020	Weak genetic differentiation	26 codons	Codon 372	Undetected
*Boleophthalmus pectinirostris*	010010	Strong genetic differentiation	50 codons	Undetected	Undetected
*Ammodytes personatus*	010020	Strong genetic differentiation	143 codons	Undetected	Codon 352, 371
*Pampus argenteus*	010010	Strong genetic differentiation	100 codons	Codon 320, 374	Codon 4, 14, 158, 214, 233, 240, 246, 320, 327, 356, 365, 366, 372, 376
*Mugil cephalus*	010010	Strong genetic differentiation	42 codons	Undetected	Codon 3, 234, 239, 303, 320, 323, 369, 372

Note: ^1^ sites were detected under purifying selection in at least one selection test; ^2^ sites were detected under positive selection in at least two selection test; and ^3^ sites were non-synonymous substitutions and fixed in certain branches.

It is worth noting that codon 372 and its neighbors in cytb gene are likely favored in adaptation in the reviewed species. Codon 372 was identified as an adaptive site in four out of six species (codon 314 in *P.* sp.1 and codon 352, 371 in *A. personatus*) (Table 2). In *E. encrasicolus*, codon 368 was also identified as an adaptive site (Silva et al. 2014). Furthermore, interspecific adaptive evolution analyses were also implemented based on cytb gene sequences of *L. polyactis* (GenBank accessions: FJ609001–609019) and *L. crocea* (GenBank accessions: EU346914–346932). Similarly, we detected codon 372 as one of the fixed adaptive sites between the two closely related species. It should be noted that the individuals collected in Sun et al. (2012) possibly contained more than one valid *Pampus* species (Li et al. 2019). Therefore, the detected positively adaptive sites in *Pampus argenteus* herein should be considered as interspecific adaptive sites, mirroring the results between *L. polyactis* and *L. crocea*. Our previous study revealed codon 372 of cytb gene in *S. marmoratus* was situated in the last transmembrane domain, which is functionally important in the energy metabolism pathways (Xu et al. 2017). For the interspecific comparison between *L. polyactis* and *L. crocea*, as *L. crocea* is better adapted than *L. polyactis* in warmer environments (Xiao et al. 2009; Xu et al. 2018), the detected interspecific substitutions might be also associated with the thermal adaptation of *L. crocea*. Therefore, codon 372 of cytb gene and its neighboring sites might be associated with metabolic processes and play important roles in thermal adaptation. Although the reviewed species are distantly related, they showed similar or identical adaptive sites in the mitochondrial cytb gene, suggesting potential genetic convergence. However, no evidence of convergent nucleotide or amino acid substitution was detected. More species and evidence (e.g., RNA-level gene expression evidence) are warranted to further confirm potential adaptive convergence. Compared to positively adaptive sites, the relatively high levels of purifying selected sites might be due to the strict functional constraints of the mitochondrial cytb gene (Sun et al. 2011), suggesting relaxed purifying selection in mitochondrial cytb gene sequences. Large numbers of genetic variations can provide fundamental sources for the evolution of population differentiation and reproductive isolation (Schluter and Conte 2009). This might be an alternative reason as to why relatively more purifying selected sites were detected in the reviewed species.

Adaptive evolution is ubiquitous. Due to environmental deviation, intraspecific differentiation would arise in distinct populations to adapt to the local environment. Adaptive evolution may also generate barriers to population connectivity and ultimately lead to further ecological differentiation (Nosil et al. 2009). For instance, strong reproductive isolation was detected in European flounders (*Platichthys flesus*) in the Baltic, which exhibit rapid ecological speciation due to salinity adaptation (Momigliano et al. 2017). However, evidence for ecological speciation in the marine realm is scarce, especially associated with reproductive isolation (Momigliano et al. 2017). In the present study, the reviewed species revealed serial stages of genetic divergence: from panmixia to strong population differentiation. By integrating genetic background information and the results of adaptive analyses, we tentatively identify four types of adaptive patterns of the reviewed species, from weak genetic differentiation to strong genetic differentiation (Table 3). It is plausible that adaptive evolution probably played an important role in maintaining and facilitating population differentiation, particularly in one of the reviewed species, *A. personatus*, of which different evolutionary lineages were sympatrically distributed (Han et al. 2012; Deng et al. 2019). Further population genomic and transcriptomic approaches are warranted to assess genome-wide adaptive patterns and demographic histories of these species.

**Table 3.** Summary of the four types of adaptive patterns of the reviewed species in this study.

	Genetic background	Adaptive pattern	Examples in this study
Type I	weak and non-significant genetic differentiation	Undetected	*Larimichthys polyactis*
Type II	weak but significant genetic differentiation	Detected	*Sebastiscus marmoratus*, *Platycephalus* sp.1, *Trachidermus fasciatus*
Type III	synonymous substitution inducted strong genetic differentiation	Undetected	*Boleophthalmus pectinirostris*
Type IV	non-synonymous substitution induced strong genetic differentiation	Detected	*Ammodytes personatus*, *Pampus argenteus*, *Mugil cephalus*
